# The role of human rights litigation in improving access to reproductive health care and achieving reductions in maternal mortality

**DOI:** 10.1186/s12884-017-1496-0

**Published:** 2017-11-08

**Authors:** Jennifer Templeton Dunn, Katherine Lesyna, Anna Zaret

**Affiliations:** 1 University of California Hastings College of the Law, San Francisco, CA USA; 2 Global Health Sciences, University of California San Francisco, San Francisco, CA USA; 3 UCSF/UC Hastings Consortium on Law, Science & Health Policy, San Francisco, CA USA

**Keywords:** Human rights, Litigation, Maternal mortality, Reproductive health care, International law, Constitutional law

## Abstract

**Background:**

Improving maternal health, reducing global maternal mortality, and working toward universal access to reproductive health care are global priorities for United Nations agencies, national governments, and civil society organizations. Human rights lawyers have joined this global movement, using international law and domestic constitutions to hold nations accountable for preventable maternal death and for failing to provide access to reproductive health care services.

**Case presentation:**

This article discusses three decisions in which international treaty bodies find the nations of Brazil and Peru responsible for violations of the Convention on the Elimination of All Forms of Discrimination Against Women and the International Covenant on Civil and Political Rights and also two domestic decisions alleging constitutional violations in India and Uganda.

**Conclusions:**

The authors analyze the impact of these decisions on access to maternal and other reproductive health services in Brazil, Peru, India, and Uganda and conclude that litigation is most effective when aligned with ongoing efforts by the public health community and civil society organizations. In filing these complaints and cases on behalf of individual women and their families, legal advocates highlight health system failures and challenge the historical structures and hierarchies that discriminate against and devalue women. These international and domestic decisions empower women and their communities and inspire nations and other stakeholders to commit to broader social, economic, and political change. Human rights litigation brings attention to existing public health campaigns and supports the development of local and global movements and coalitions to improve women’s health.

**Electronic supplementary material:**

The online version of this article doi: (10.1186/s12884-017-1496-0) contains supplementary material, which is available to authorized users.

## Introduction

Human rights litigation can inspire the transformation of social norms and play a critical role in supporting domestic programs and campaigns designed to improve access to reproductive health care and reduce maternal mortality. Over the last decade, United Nations (UN) agencies, national governments, and civil society organizations (CSOs)^1^ collectively prioritized the need to improve maternal health, reduce global maternal mortality, and work toward universal access to reproductive health care [1]. Similarly, human rights lawyers have turned to international law and domestic constitutions to address barriers in accessing reproductive health care services and hold state governments accountable for preventable maternal death [2–14]. This article analyzes the impact of international and domestic decisions on access to reproductive health care and discusses how human rights litigation can play a role in supporting existing campaigns and mobilizing new movements that seek to improve access to reproductive health services and address the tragically high rate of preventable maternal death.

Human rights litigation has an additional function beyond the practical impact on access to a particular health service or even the broader goal of producing health system improvements. “Human rights litigation … is a specific form of litigation centered on promoting structural and systemic changes in order to bring about social transformations and address unequal power relations” [15]. Law has an indisputable influence on social and cultural systems, structures, and hierarchies. Laws and social norms can interact to disempower women, or they can be used to empower women (for example, laws granting or restricting the right to vote, to pursue an education, or to own property). Human rights laws work to empower individuals and communities by promising “equal power to each person, including persons who would otherwise be powerless” [16]. In bringing litigation based on human rights principles into national and international courts, legal advocates highlight the disempowerment of individual women in order to address systemic discrimination and inspire nations and other stakeholders to commit to broader social, economic, and political change. In the case studies discussed below, national courts and international treaty bodies challenge historical structures and hierarchies that discriminate against, devalue, and disempower women, illustrating how human rights litigation has become an empowering strategy over the last decade. In bringing issues that impact women, such as abortion and maternal mortality, out of the private sphere and into the public sphere, litigation deconstructs socio-political structures where women’s health is minimalized, maternal death is normalized, and women’s lives are devalued.

Addressing barriers to achieving universal access to reproductive health, including perinatal care and safe abortion, is a public health priority warranting global attention and demanding multifaceted approaches and inclusive, collaborative solutions. A discussion of these barriers and why reproductive and maternal health is a global priority is the focus of the next section of this article. Then the following section provides a brief introduction to international law and describes how UN treaty bodies implement and enforce international treaties. This part discusses three international decisions where treaty bodies held nations accountable for failing to provide access to quality, non-discriminatory perinatal care, and legal abortion. This section also evaluates the impact of these cases and discusses the strengths and limitations of using international law as a tool to promote access to reproductive health care. Subsequently, we present a section on domestic litigation, analyzing two maternal health cases brought before domestic courts in India and Uganda. In these cases, the litigants relied on national constitutions to demand that their governments provide adequate maternal and reproductive health care. These domestic cases demonstrate how litigation intersects with and complements political and social movements and provides momentum to effectuate change. The following section summarizes the limitations in using human rights litigation to improve reproductive and maternal health and discusses how CSOs can address or ameliorate some of these shortcomings. In addition, this section proposes ways in which human rights litigation can further support efforts from UN agencies, CSOs, and other stakeholders to implement broader health system improvements going forward. This article concludes by emphasizing that the significance of court and treaty body decisions extends beyond the provision of practical recommendations to improve women’s health. Law has a norm-setting function; human rights litigation can be used to address the social, political, and legal structures that have historically disempowered and devalued women. By challenging unjust social, cultural, and political inequities and the systemic discrimination that constrain women’s access to reproductive health care and contribute to preventable maternal death, domestic courts and international treaty bodies effectively transform these discriminatory norms and affirm the value of women’s lives.

## Background

Over the last decade, UN agencies, national governments, and CSOs have committed to the shared goals of improving access to reproductive health care and reducing maternal mortality [1]. As a result of a concerted effort by these organizations to develop a strategic, focused approach to maternal health and mortality, the maternal mortality ratio decreased by approximately 44% between 1990 and 2015, from 385 to 216 deaths per 100,000 live births [17]. Despite these improvements, approximately 303,000 women still died during childbirth in 2015, with 99% of these deaths occurring in developing countries [17, 18]. While the number of maternal deaths remains high in many regions of the world, the majority of these deaths are preventable.

In addition to reducing global maternal mortality, access to reproductive health care services improved from 1990 to 2015. Efforts by the international community have resulted in increases in the number of women in developing regions receiving the recommended four antenatal care visits (35 to 52%), the proportion of deliveries worldwide with a skilled birth attendant (59 to 71%), and the global contraceptive prevalence rate (55 to 64%) [1]. These efforts have also decreased the global adolescent birth rate (59 to 51 births per 1000 girls) and the unmet need for family planning (15 to 12%) [1]. Even with this progress, large inequities exist across and within regions, and millions of women worldwide lack access to basic reproductive health care services, including safe abortion.

Access to safe abortion remains a public health concern worldwide. Due to laws restricting and criminalizing abortion [19], many women are forced to seek unsafe abortions or carry out a pregnancy, even where the pregnancy poses risks to the mother’s life or health. In addition to the significant implications on women’s privacy, dignity, and right to make autonomous decisions, laws criminalizing abortion have an adverse impact on women’s health. Unsafe abortion accounts for an estimated 13% of global maternal deaths, the majority of which occur in countries with more restrictive abortion laws [20]. In addition, in 2012 approximately 6.9 million women in developing regions were treated for complications from unsafe abortions [21], and it is estimated that an additional 40% of women with complications never receive treatment [22]. Improvements in access to reproductive health care services, including increased access to family planning, can help reduce unintended pregnancies, unsafe abortion, and maternal deaths [1].

In 2015, the UN and international community renewed their commitment to reducing maternal mortality and ensuring universal access to reproductive health care services with a new focus on integrated, interdisciplinary solutions and inclusive, collaborative approaches [23–25]. The World Health Organization (WHO), the UN agency tasked with directing and coordinating global health, called on the public health community to develop “broader, cross-cutting approaches coordinated around and aligned with countries’ needs and priorities” [24]. Human rights litigation highlights the disempowerment of individual women, calls on governments to address systemic discrimination, and inspires commitment to broader social, economic, and political change. Therefore, litigation can play a crucial role in setting these types of “broader, cross-cutting” changes in motion. Litigation should, however, be viewed as one tool for implementing broader systemic change, which ultimately requires focusing on the realities facing vulnerable and marginalized groups and building multifaceted collaborations within the health sector and beyond.

## Case presentations

The following sections explore how human rights litigation can help achieve the global goals of improving access to reproductive health care services and reducing preventable maternal death.

## The right to health under international law

### Sources for the right to health under international law

In order to hold a nation accountable for human rights violations, the nation must first “agree” to be bound by international law by ratifying a treaty [26]. Treaties (also called “covenants” or “conventions”) are formal agreements between countries. When a treaty is adopted by the General Assembly of the UN, it creates legally binding international obligations for the member states that sign and ratify the treaty [26]. Another less common means of holding states accountable for human rights violations is by alleging that the nation violated “international customary law” [27]. Customary laws are norms that have become so ingrained in domestic and international laws that countries need not consent in order to be bound [26, 27].

UN treaty bodies (also called “committees”) are responsible for monitoring whether a country is violating a treaty. Monitoring involves issuing reports about compliance with treaties, receiving country reports, issuing decisions, and, in some cases, receiving individual or group complaints about treaty violations [26]. While treaty bodies do not have traditional police power to enforce a decision on an individual complaint, nations generally comply with treaty body recommendations. However, as demonstrated in the cases below, implementation is frequently a gradual rather than an immediate process and may take persistent pressure from the treaty body or CSOs to ensure compliance.

Several treaties specifically address the government’s obligation to provide non-discriminatory access to health services, including family planning, prenatal care, and other reproductive health care [28, 29]. This article will focus on three cases, brought under the Convention on the Elimination of All Forms of Discrimination Against Women (CEDAW) and the International Covenant on Civil and Political Rights (ICCPR).

CEDAW is often described as the “international bill of rights for women” [28]. Human rights advocates have successfully used provisions in CEDAW to advocate for the non-discriminatory provision of health care services, including the right to access reproductive health services [2, 4, 5]. Countries which have ratified CEDAW commit to taking “all appropriate measures” to eliminate discrimination against women in the “political, economic, social, cultural, civil or any other field” [28]. This promise includes ensuring that women have the right to vote and hold elected office, the right to pursue an education, and the right to own property [28]. CEDAW does not specifically include the right to health; however, it does provide protection for pregnant women and includes provisions that address the right to access reproductive and maternity care [28]. Article 12 of CEDAW contains specific provisions calling on states to guarantee access to family planning and perinatal care:

States Parties shall take all appropriate measures to eliminate discrimination against women in the field of health care in order to ensure, on a basis of equality of men and women, access to health care services, including those related to family planning … States Parties shall ensure to women appropriate services in connection with pregnancy, confinement and the postnatal period, granting free services where necessary, as well as adequate nutrition during pregnancy and lactation [28].

This strong language supporting women’s health enabled the Committee on the Elimination of Discrimination against Women (CEDAW Committee) to be the first international treaty body to hold a national government responsible for preventable maternal death. In *Alyne v. Brazil*, discussed below, the CEDAW Committee determined that the failure of the Brazilian public health system to provide non-discriminatory health services to Alyne da Silva Pimentel Teixeira violated her rights to health and non-discrimination [2].

The ICCPR is an international human rights treaty which commits nations to protecting individual civil and political rights, and is monitored by the UN Human Rights Committee [30]. The ICCPR provisions, particularly the right to life, have served as a tool for human rights litigators and CSOs working to ensure the right to maternity care and access to reproductive health care services [2, 3]. Although the ICCPR does not contain specific references to the right to health, there is a close relationship between the right to health and the civil and political rights specifically enumerated in the ICCPR, such as the right to human dignity, the right to life, equality and non-discrimination, privacy, and the prohibition against torture [30]. The ICCPR’s protections for the right to life have also been interpreted broadly to include the right to “maternal health” [31].

Although the Human Rights Committee is the implementing treaty body for the ICCPR, as shown below in the analysis of case law, other international treaty bodies [2] and domestic courts [12] frequently reference and rely on the ICCPR’s provisions and protections to support findings of human rights violations. The following case studies from Brazil and Peru examine successful international human rights litigation involving maternal health and mortality in order to demonstrate the strengths and limitations of using international litigation to achieve maternal and reproductive health goals.

### Landmark CEDAW decision on intersectional discrimination and preventable maternal mortality in Brazil

In 2011, the CEDAW Committee issued two historic decisions involving the government’s responsibilities with respect to maternal health and abortion. In the first decision, *Alyne da Silva Pimentel Teixeira v. Brazil* (“*Alyne v. Brazil”* or “*Alyne* decision”), the CEDAW Committee became the first international treaty body to hold a nation accountable for preventable maternal death [2, 32].

Alyne da Silva Pimentel Teixeira was a pregnant Brazilian woman of African descent. When Alyne was 6 months pregnant, she sought care for nausea and abdominal pain in a private health clinic contracted with Brazil’s government-run and publicly financed health system [2]. She was treated and returned home, but she returned to the clinic 2 days later after her symptoms had worsened [2]. No fetal heartbeat was detected at that time, and Alyne was given medication to induce the delivery of the stillborn fetus [2]. A dilation and curettage procedure was also performed at the clinic to evacuate her uterus and remove retained parts of the placenta and afterbirth [2].

As Alyne remained under observation, her condition worsened [2]. She began hemorrhaging and suffering from other complications of the stillbirth [2]. The clinic doctors recommended transfer to a better equipped public hospital [2]. However, the public hospital refused to use its only ambulance to transport her, and her family was unable to obtain a private ambulance [2]. Alyne waited 8 hours in critical condition before reaching the hospital [2]. Once transferred, she was left largely unattended in the hospital hallway for 21 hours until she died [2].

Alyne’s family brought a claim against the Brazilian government, asserting violations of CEDAW and other international and domestic laws. In 2011, the CEDAW Committee ruled in favor of Alyne’s family. In rendering its decision, the Committee emphasized that Afro-Brazilian women are seven times more likely than other Brazilian women to die during pregnancy and childbirth [2, 33]. After reviewing the record, the CEDAW Committee determined that Alyne had died of entirely preventable causes because of her race, gender, and economic status. Thus, the Committee concluded that Alyne had experienced “intersectional discrimination” as she was “discriminated against, not only on the basis of her sex, but also on the basis of her status as a woman of African descent and her socioeconomic background” [2]. The CEDAW Committee found that Alyne’s death constituted gender discrimination under CEDAW and a violation of the right to life under the ICCPR [2].

The Committee’s decision assessed the realities facing Brazilian women of African descent and other marginalized and vulnerable groups, such as indigenous, ethnic, or other minority populations in Brazil. These groups often receive lower quality health care [34] and have significantly higher risks of maternal mortality [33, 35, 36]. While Brazil has reduced the national maternal mortality ratio by approximately 57.7% from 104 to 44 deaths per 100,000 live births over the past 25 years [17], stark health disparities exist between populations. The court’s attention to realities such as these paved the way for further actions and dialogues addressing socioeconomic discrimination and the interaction between race, sex, and economics.

As the first case of an international treaty body to hold a government accountable for preventable maternal death, *Alyne v. Brazil* brought global attention to the issue and influenced both international [5] and domestic courts [11, 37] charged with reviewing sex, race, and socioeconomic discrimination, and related health system disparities. The decision has also been the subject of discussion and analysis among legal scholars as a groundbreaking model for using human rights litigation to hold governments accountable for gender-based discrimination in health care and preventable maternal mortality [15, 32, 38, 39].

While the decision set a strong legal precedent for protections for maternal mortality under a human rights framework, implementing the CEDAW Committee’s recommendations has presented challenges at the national level. Shortly after the CEDAW decision, Brazil set up an interministerial working group with members of the Ministry of Foreign Affairs, Ministry of Health, Secretariat for Women’s Policies, Secretariat for Human Rights, and the Secretariat for the Promotion of Racial Equality to implement the recommendations from the decision [40]. The working group was tasked with developing a plan for complying with CEDAW’s decision and monitoring of a new maternal and reproductive health program [41]. Brazil also settled on an amount to compensate Alyne’s family. However, in 2013, 2 years after the CEDAW decision, the Brazilian government had not addressed the health system failures that led to Alyne’s death or compensated Alyne’s family.

After this 2-year period passed without meaningful implementation of the recommendations, international and regional CSOs demanded that Brazil take action. In 2013, Brazil’s Senate convened hearings to elicit testimony on the government’s progress in meeting the goals set by the working group. Armed with the CEDAW decision, CSOs presented documentation and testimony and called on the government to compensate Alyne’s family and address the pervasive discrimination in Brazil’s health system and the persistence of poor health facility conditions [42–44]. In 2014, Brazil finally paid reparations to Alyne’s mother [45, 46]. Although Brazil acknowledged its responsibility in Alyne’s death and made some inroads toward implementing a new maternal and reproductive health program [41], the nation’s efforts to address the broader health system challenges and meet the goals of the working group are still ongoing.

The events that followed the *Alyne* decision demonstrate that CSOs and other stakeholders can use human rights litigation as a political tool to demand that a nation fulfill its human rights obligations. The decision in *Alyne v. Brazil* brought public awareness within Brazil about the pervasive discrimination within the health system and provided leverage for CSOs to call on the Brazilian government to finally implement the broader health system changes recommended by the CEDAW Committee. However, women in Brazil continue to face discrimination, and stark health disparities persist between populations. The continued and persistent effort by international, national, and regional stakeholders is crucial to progress from the promise of the right to health to the realization of the right.

### Using the ICCPR and CEDAW to address obstacles in accessing legal abortion in Peru

In Peru, international treaty monitoring bodies have issued two major decisions regarding Peruvians’ right to access legal abortion. The first of these decisions, *Karen Noelia Llantoy Huaman v. Peru* (“*K.L. v. Peru*”), was issued in 2005 by the UN Human Rights Committee. In *K.L. v. Peru*, medical providers denied a 17-year-old girl an abortion even though her physicians had determined that the fetus was anencephalic and would not survive past childbirth [3]. The Human Rights Committee found that Peru’s abortion law entitled K.L. to a legal abortion [3]. The Committee then concluded that Peru had violated international and Peruvian law by not providing a procedural mechanism to effectuate the right [3]. The Human Rights Committee determined that Peru’s failure to provide a mechanism for accessing legal abortion under the exceptions violated the ICCPR and other international and domestic laws [3].

In 2011, the CEDAW Committee decided *L.C. v. Peru*, the second major decision by the CEDAW Committee on the right to maternal health and abortion, and the second decision by any international treaty monitoring body specifically addressing those rights in Peru. In *L.C. v. Peru*, medical providers in Peru denied a 13-year-old girl access to abortion and also denied her access to medically necessary back surgery because she was pregnant, which caused her to become permanently paralyzed [4]. A 34-year-old man had repeatedly raped L.C. over a period of several months, resulting in pregnancy [4]. After becoming pregnant, L.C. attempted suicide by jumping off a building, severely injuring her spine [4]. Her physicians recommended immediate surgery to repair her spine, which the hospital refused to provide, stating that back surgery would interfere with her pregnancy [4]. L.C.’s mother then asked the hospital to authorize an abortion [4]. The hospital feared prosecution under Peru’s criminal abortion law and, therefore, refused to allow the abortion [4]. L.C. suffered a miscarriage, after which the hospital authorized the spinal surgery [4]. However, the surgery was performed too late to avoid permanent damage to her spine, and, as a result, L.C. was permanently paralyzed from the neck down [4].

The penal code restricting abortion in Peru allows abortion in order to “save the life of the woman or to avoid serious and permanent damage to her health” [47]. However, at the time L.C. sought abortion care and back surgery, the government did not provide guidance on how to interpret the language of the statute [47]. Despite repeated requests and appeals, Peru’s Ministry of Health provided no guidelines for physicians or hospitals on when they could legally provide abortion, or on what constitutes “serious” damage to one’s health. The vagueness of the criminal abortion statute had a chilling effect on physicians, who feared arrest and prosecution [48]. In addition, many women were unaware that therapeutic abortions were permitted under the law [48]. This resulted in cases like L.C.’s, in which medical providers denied abortions even where authorized under Peru’s limited health exception.

In response to Peru’s failure to provide clear guidance to medical providers about when abortion can be legally provided, human rights lawyers filed a complaint under CEDAW. The lawyers argued that L.C.’s abortion was legally authorized and should have been provided under the maternal health exception to Peru’s criminal abortion law. The CEDAW Committee determined that Peru had violated CEDAW provisions that require governments to end discrimination against women, provide equal protection under the law, protect women’s human rights, and specifically to eliminate gender-based discrimination in health care services [28]. As the Committee explained,

[O]wing to her condition as a pregnant woman, L.C. did not have access to an effective and accessible procedure allowing her to establish her entitlement to the medical services that her physical and mental condition required. … This is even more serious considering that she was a minor and a victim of sexual abuse, as a result of which she attempted suicide. The suicide attempt is a demonstration of the amount of mental suffering she had experienced [4].

The Committee concluded that Peru failed to ensure access to therapeutic abortion and essential health care services as required under CEDAW and Peruvian law [4].

Despite the two decisions, Peru did not immediately act to implement the recommendations in *K.L. v. Peru* and *L.C. v. Peru*. In response to the lack of action, public health advocates, doctors, and lawyers began a continuous and concerted effort to demand that Peru implement the recommendations by the Human Rights Committee and the CEDAW Committee [49]. In 2014, in response to this interdisciplinary pressure, Peru finally developed and published hospital guidelines [50] for accessing legal abortion based on maternal health indications. In 2015, K.L. was finally compensated [51], and in March 2016, Peru formally apologized to L.C., acknowledging that the government and public health system had failed her [52].^2^

The 2005 decision in *K.L. v. Peru* served as a catalyst for further advocacy in Peru. However, the case demonstrates that a favorable decision alone will not bring about an immediate transformation. Indeed, change in Peru only materialized after decisions by two separate international treaty bodies and the persistent dedication of CSOs and other stakeholders. The eventual adoption of the new guidelines and Peru’s public acknowledgement of the gravity of the human rights violations against K.L. and L.C. was a historic first step in addressing the connection between access to safe abortion and the government’s responsibility for ensuring women’s health in Peru. The ongoing political engagement also resulted in the publication of a 2016 report by the UN Committee on the Rights of the Child, calling on Peru to decriminalize abortion and postabortion care in all circumstances, not only for maternal health indications [53].

Similar to the *Alyne* decision, the *K.L.* and *L.C.* decisions serve as significant precedents for holding governments accountable for the failure to ensure access to necessary reproductive health care services. Moreover, these decisions galvanized broader attention to the government’s role in ensuring safe and accessible maternal and reproductive health care services.

## The right to health under national constitutions

Two thirds of national constitutions have a provision addressing the right to health [54]. These constitutional provisions express a national commitment to promoting health and often accompany an intent to provide or facilitate health care services for the entire population. The right to health within constitutions and other domestic law provides support for human rights lawyers and CSOs to advocate “for better health and health care as well as for implementation of the international human right to health” [54]. In the following two cases, human rights attorneys brought claims in domestic courts based on the government’s failure to provide adequate maternal and reproductive health care to women in violation of the constitutional right to health. The impact of the cases transcended the claims brought by individual plaintiffs and their family members, providing momentum for human rights lawyers, CSOs, and other stakeholders to demand improvements in the delivery of public health care services and other governmental benefits relating to reproductive and maternal health.

### Pursuing the right to maternal health care and reproductive rights through domestic litigation in India

In the consolidated decision, *Laxmi Mandal v. Deen Dayal Harinagar Hospital & Others & Jaitun v. Maternal Home MCD, Jangpura & Others* (“*Laxmi Mandal*”)*,* the High Court of Delhi reviewed claims that public health services failed two pregnant women by denying them health care and other services to which they were entitled under India’s existing health care and social services programs [12].

The first case addressed in the decision involved Shanti Devi, an Indian woman who died shortly after the premature birth of her sixth child [12]. Shanti Devi had experienced complications during earlier pregnancies and several obstacles in accessing necessary medical care [12]. After experiencing a stillbirth in 2008 during her fifth pregnancy, she was unable to access and obtain the care she needed [12]. Although she lived below the poverty line and qualified for public benefits, four separate public hospitals refused to admit her because she did not have a ration card and could not demonstrate her eligibility for free health services [12]. Shanti Devi attempted to access care in several facilities over a 2-week period, and she was finally admitted to a public hospital in Delhi for removal of the stillborn fetus [12]. She was released shortly after despite her need for continued medical attention [12]. Her brother-in-law filed a writ petition on her behalf [55], and with the assistance of the Human Rights Law Network (HRLN), she was readmitted to the hospital for 18 days [56]. When she was released from the hospital, she was not provided family planning counseling, access to contraceptives, or follow-up care as required under government programs [12]. When Shanti Devi became pregnant with her sixth child, she did not go to the hospital for prenatal care, fearing she would face similar obstacles [12]. She went into premature labor at home, without a physician, skilled birth attendant, or any other medical attention, and she died as a result of complications that were entirely treatable and preventable [12].

The second case addressed in the decision involved Fatema, a homeless and pregnant woman suffering from anemia, epilepsy, and other conditions that put her at higher risk during her pregnancy [12]. Fatema made several visits to government health facilities and shelters and was denied medical care and other assistance guaranteed under existing government programs [12]. After being denied admittance to the hospital and turned away from the maternity home, Fatema gave birth to her baby Alisha under a tree, in full public view [12].

In its decision, the Delhi High Court found that Shanti Devi and Fatema were denied public benefits to which they were entitled and that the public health system failed them both in violation of the right to life and health under the Constitution of India [12]. The court based its decision on the right to health first expressed in *Paschim Banga Khet Mazdoor Samity & Ors v. State of West Bengal & Anor*. In that case, the Supreme Court of India held for the first time that the right to life in the constitution included an obligation to provide timely medical treatment necessary to preserve human life [57]*.* Following the *Paschim* decision, the court determined that the government’s failure to act to protect and care for Shanti Devi and Fatema violated the constitution [12].

With the second highest number of maternal deaths globally in 2015 and a maternal mortality ratio of 174 deaths per 100,000 live births [17], India struggles with the implementation of their various maternal health programs. Low levels of awareness regarding programs to benefit pregnant women [58–60], administrative barriers that inhibit access to the benefits or cause increased costs [61, 62], and the poor quality of emergency obstetric care services [63, 64] all contribute to the struggles of implementing the programs and improving access and quality of necessary health services in India. These barriers and existing health system inequities are exacerbated by discrimination based on education, socioeconomic status, and the caste system [64]. Despite the challenges in implementing the existing programs, India continues to develop new maternal health programs in an attempt to combat their high maternal mortality ratio [65].

The *Laxmi Mandal* decision exposed the failures in implementing India’s health programs and empowered a domestic campaign to ensure access to safe motherhood and reproductive health care services. The decision became the subject of substantial review by legal scholars [56, 66] and influenced subsequent judicial decisions in India and other countries [11, 66, 67]. In addition, the decision set a national and international precedent for using constitutions to support maternal health rights and provided advocates with an additional tool for holding governments accountable.

With the *Laxmi Mandal* decision in hand, lawyers and CSOs have continued to fight for universal access to maternal health care services and other government programs [66, 67]. After the decision, the HRLN, the organization that brought *Laxmi Mandal*, demanded implementation of the court orders and filed 25 additional cases that address other aspects of maternal health in the country [66, 68]. Courts also intervened *sua sponte* to enforce the decision’s directives upon hearing accounts of women’s failures to access India’s various family planning and maternal health care schemes [67].

The *Laxmi Mandal* decision thus brought attention to the difficulties in implementing India’s multiple maternal health programs within a fragile health system and the government’s failure to reach the most vulnerable populations. Community rights awareness trainings, fact-finding missions, and further litigation by HRLN and other human rights lawyers have kept the conversation surrounding the challenges in implementing existing maternal health programs ongoing. Continued and persistent efforts of CSOs, legal organizations such as HLRN, and the public health sector are needed to ensure implementation of these programs and demand accountability from the government. The concerted efforts of these various stakeholders could bring India closer to the broader systemic change that India desperately needs.

### How a negative decision in Uganda’s constitutional court became the catalyst for addressing preventable maternal death and mobilizing for broader health system change

Petition No. 16, a landmark case in Uganda, was filed in 2011, demanding that the government provide the essential maternal health commodities and quality health services necessary to reduce the high rate of maternal mortality in Uganda. The case involved two women, Sylvia Nalubowa and Jennifer Anguko, who both died during childbirth. In 2009, Sylvia Nalubowa went into labor at a local health center [37]. After Nalubowa had delivered her baby, the midwife realized that she was having twins and required emergency obstetric care not available at the health center [37]. Nalubowa was referred to the district hospital to deliver the second baby [37]. Nurses at the hospital asked Nalubowa to pay for maternity services and commodities before she could receive treatment [37, 69]. In extreme pain and bleeding profusely, Nalubowa promised her land in exchange for medical care [37]. Tragically, instead of receiving medical attention, Nalubowa died with one of the twin babies still in her womb [37]. In 2010, Jennifer Anguko, a pregnant woman experiencing a health emergency arrived at a public hospital [37]. She did not receive care for more than ten hours, despite her husband’s attempts to gain the attention of the nurses [37]. By the time Anguko finally received medical attention and was taken to surgery her uterus had ruptured and it was too late [69]. Anguko and her baby both died [69].

In Uganda, stories like Anguko’s and Nalubowa’s are not uncommon. Uganda is among one of ten countries that account for 60% of the number of global maternal deaths, with maternal mortality ratio estimates ranging from 343–435 deaths per 100,000 live births [17, 70]. Despite efforts to make health care services accessible to Ugandans by abolishing user fees in 2001, insufficient resources in Uganda’s public health system mean that pharmaceuticals are scarcely available, health care workers are frequently absent, and patients may be required to pay “informal fees” before they can receive health services [71, 72]. These health system challenges are exacerbated by “discriminatory social practices,” “negative attitudes towards women and girls,” and the “limited power women and girls have over their reproductive lives” [37]. The combination of limited health care resources and the disempowerment of women and girls has a significant impact on the quality, affordability, and accessibility of health services for women in Uganda and directly influences maternal mortality [71, 72].

In 2011, lawyers at the Centre for Health, Human Rights & Development (CEHURD) filed Petition No. 16 in the Constitutional Court of Uganda against the Ugandan government based on the failure to prevent the pregnancy-related deaths of Sylvia Nalubowa and Jennifer Anguko. CEHURD argued that maternal health services and commodities, including a birthing kit, should be provided in government health facilities free of charge and further that Uganda violated international and constitutional law by not providing this “basic maternal health care package” to Nalubowa and Anguko, as well as hundreds of Ugandan women in similar circumstances [13].

The Attorney General of Uganda argued that the complaint’s allegations required the court to make a judicial decision on a “political question” involving state priorities and budget allocation best left to the legislative or executive branches of government [13]. The Constitutional Court agreed and dismissed the case based on the political question doctrine, stating that the court has “no power to determine or enforce its jurisdiction on matters that require analysis of the health sector government policies… If this Court determines the issues raised in the petition, it will be substituting its discretion for that of the executive granted to it by law” [13]. Plaintiffs appealed the decision to the Supreme Court of Uganda.

In 2015, the Supreme Court of Uganda reversed the Constitutional Court’s decision and held that the Ugandan Constitution provided direct access to the Constitutional Court for constitutional interpretation and that the political question doctrine did not apply to the constitutional claims asserted in the case [14]. In his concurring opinion [14], Chief Justice Katureebe directed the Constitutional Court’s attention to two cases, *Minister of Health and Others v. Treatment Action Campaign of 2002* [73] and *Paschim Banga* [57], wherein the Constitutional Court of South Africa and the Supreme Court of India found a right to health in their state constitutions. Thus, in addition to rejecting the application of the political question doctrine and remanding the case to the constitutional court for consideration, the Chief Justice also provided the Constitutional Court with direction on how to assess the merits of the petitioners’ constitutional challenge [14]. The Supreme Court directed the Constitutional Court to consider whether the facts in the case were supported by evidence, and if so, to determine whether failure to deliver maternal health care services violated the right to access medical services under the constitution [14]. On September 1, 2016, the case was reopened by the Constitutional Court in Uganda and is currently pending [74].

Even though the Constitutional Court decided against the plaintiffs, the case brought public attention and advocacy to the issue of maternal mortality in Uganda. After Petition No. 16 was filed, various CSOs working independently on maternal health issues created the Coalition to Stop Maternal Mortality [75]. In 2012, the Coalition, other CSOs, and the public health community advocated for an increase in the budget to recruit, motivate, and retain health care workers [75]. They successfully encouraged members of Parliament to block a new budget that failed to address the Coalition’s demands. As a result, members of Parliament and the President agreed to allocate approximately $15 million dollars to address the health care workforce shortage [75–77]. Petition No. 16 provided the Coalition and partnering CSOs with a focal point and support for their campaign.

Petition No. 16 and the tragic stories of Sylvia Nalubowa and Jennifer Anguko sparked extensive media coverage and focused public attention on the prevalence of maternal death in Uganda and the need to address a government health system that is rife with social and cultural hierarchies that discriminate against, devalue, and disempower women. Even before the Constitutional Court’s decision on Petition No. 16, the Coalition mobilized the public to attend court hearings and pressure the government to move forward with the case [75]. After the Constitutional Court’s decision, the dismissal of the case was used as a mobilizing force for international and national CSOs to demand that parliament direct more resources into Uganda’s struggling public health system [75]. The decision also brought support and resources into Uganda to help local CSOs pursue the appeal and obtain the subsequent successful ruling by the Supreme Court of Uganda. The combined efforts of human rights lawyers and other actors resulted in strategic, targeted efforts which amplified the potential impact any single actor or advocacy group could have made alone.

## Discussion

While human rights litigation can be a powerful force for change, practical limitations persist. A specific enforcement mechanism for international decisions does not exist, and ensuring implementation of treaty body recommendations is often difficult. The decision may have little or no immediate impact for years, as illustrated by the decade that passed between the decision in *K.L. v. Peru* and the government’s public acknowledgement and compensation of K.L., or even the “shorter” three years between the 2011 decision in *L.C. v. Peru* and the implementation of new abortion guidelines in 2014. In addition, very few individuals are able to have their complaints heard before an international body, such as the Human Rights Committee or CEDAW. Rather, pursuing individual claims through international litigation is often intended to influence norms and impact broader social change. Litigation can also be expensive and time-consuming, and potential benefits must be balanced against the possibility of an unfavorable decision, as seen in the outcome of Petition No. 16 in the Constitutional Court of Uganda. The potential benefits must also be balanced against the economic feasibility of persistently and repeatedly bringing individual complaints to enforce court decisions as illustrated by the *Laxmi Mandal* decision and subsequent litigation in India.

All of these cases demonstrate that human rights litigation can eventually bring about broad, systemic change; however, human rights lawyers cannot realize systemic change in isolation. Local CSOs and the public health community are integral to the successful implementation of a positive legal decision. These stakeholders all played critical roles in advocating for the government to fulfill its obligations and prioritize reproductive and maternal health.

While the human rights litigations in the case examples took different turns, they all involved commitment from CSOs and the public health community in order to realize a practical and direct impact on maternal health and the local health system. The *Alyne* decision set an international precedent and provided leverage to demand accountability for discrimination in Brazil’s health system. *K.L. v. Peru* and *L.C. v. Peru* encouraged the development of new abortion guidelines which provided clarification on the maternal health exception and increased access to legal abortion. The *Laxmi Mandal* decision exposed the implementation failures of India’s maternal health programs and provided needed support for further campaigns and litigation. In Uganda, international and local CSOs used Petition No. 16 to demand additional funding to address the health care workforce shortage in Uganda’s public health system and focus Parliament’s attention on the importance of saving women’s lives.

The decisions in these cases also brought public awareness to barriers women faced in accessing maternal and reproductive health care, and set a precedent for using human rights litigation to support the right to maternal health and accessible, quality reproductive health care services. As a result, CSOs were able to engage communities in conversations regarding maternal health issues and demand government accountability and implementation of the recommendations and court mandates. The case studies demonstrate that increasing access to maternal and reproductive health care is a gradual process, and that broad systemic change takes time and requires the combined efforts of CSOs, the public health community, and other stakeholders.

The value of litigating to protect maternal health and access to reproductive health care, however, goes beyond the practical changes that take place in response to litigation. These decisions bring attention to the plights of individual women and groups of women facing similar barriers and obstacles. In Brazil, the hospital did not send an ambulance to pick up Alyne because a Brazilian woman of African descent’s life was not considered important enough to warrant the use of the hospital’s only ambulance. After Alyne was finally transported to the hospital, she was left hemorrhaging and abandoned in the hallway for hours until she died as she was not considered worthy of priority in treatment. The CEDAW decision brought global attention to Alyne's tragic death and established norms condemning race and sex discrimination and affirming the value of the lives of all women. In finding that Brazil violated international human rights law, the CEDAW Committee acknowledged before the global community that her death was not the inevitable consequence of being female and pregnant, but instead that her death was preventable and her life mattered.

Similar parallels can be drawn to the case of Shanti Devi in India. Shanti Devi was ignored by the health system and local government programs and abandoned by the system during the first five of her six pregnancies. Administrative bureaucracy and government disregard resulted in her death during the sixth pregnancy. Like Alyne, Shanti Devi’s death was not inevitable. She died because she was a pregnant woman of lower caste and economic status who depended on government programs for prenatal and postnatal care.

Like Alyne, the government and society dismissed Shanti Devi’s life. At the time, Shanti Devi’s death was seen as a tragic but common consequence of being a pregnant woman in a country and health system that discriminates against and devalues women. The court in the Shanti Devi case held that rampant government dismissal and neglect is not simply unfortunate, but is fundamentally unconstitutional and unjust. Such decisions, whether by domestic courts or human rights treaty bodies, call on governments to account for their failure to protect vulnerable and marginalized women like Alyne and Shanti Devi, and proclaim to the nation and international community that their lives have value.

## Conclusions

Moving forward, these cases and the cases that have followed demonstrate that human rights litigation can increase access to reproductive health care and improve maternal health. International treaty bodies and domestic courts found that the governments of Brazil, Peru, Uganda, and India violated human rights laws by failing to protect women’s health and ensuring access to safe and accessible maternal and reproductive health services. With these international treaty body and domestic court decisions in hand, CSOs and the public health community called upon the government to comply with the decisions, demanding both individual reparations and the implementation of the broader, systemic changes needed to address access to reproductive health care and achieve reductions in maternal mortality. The cases studies demonstrate the importance of partnership between human rights lawyers, CSOs, and the public health community in working toward recognizing, implementing, and realizing the right to reproductive and maternal health.

Finally, by highlighting the tragic stories of Alyne da Silva Pimentel Teixeira, Karen Noelia Llantoy Huaman, L.C., Shanti Devi, Fatema, Sylvia Nalubowa, and Jennifer Anguko, human rights lawyers claimed the attention of the government and broader society. The resulting international and domestic decisions demand the government address the systemic discrimination that resulted in irreparable harm and preventable maternal death. The individual stories of these women, mothers, sisters, and wives call out for a health system and broader social and political structure where women’s health is important, women’s lives are valued, and life, rather than death, is the inevitable consequence of pregnancy and childbirth.

## Additional file

Additional file 1: Open peer review. (PDF 165 kb)

## Footnotes

1 The term “civil society organization” or CSO is defined as “the wide range of citizens’ associations that exists in virtually all member countries to provide benefits, services, or political influence to specific groups within society. CSOs include business forums, faith-based associations, labor unions, local community groups, nongovernmental organizations (NGOs), philanthropic foundations, and think tanks. Branches of government (government agencies and legislators), individual businesses, political parties, and the media are usually excluded” [78].

2 It is also important to note two limitations of the *K.L.* and *L.C.* decisions. First, neither the CEDAW Committee nor the Human Rights Committee addressed whether Peru’s criminal abortion statute violated international or domestic law. Instead, the decisions found that Peru violated international law by failing to provide a mechanism for securing a legal abortion under the exceptions to the criminal abortion law [3, 4]. Thus, Peru could have avoided implementing the decision altogether by removing the health exception or passing a new law banning abortion outright, similar to the abortion bans in nearby El Salvador, Chile, Nicaragua, Honduras, Suriname, Haiti, and the Dominican Republic. The laws in these countries either prohibit abortion altogether or do not state any explicit legal exceptions to provide an abortion to save a woman's life [79]. Finally, while the new hospital guidelines in Peru describe how to obtain a legal abortion where a pregnant woman’s life or health is at risk, the guidelines do not provide the necessary clarification concerning the treatment of a woman’s mental health under the criminal abortion statute [80, 81].
