# Supplementary material for: The role of human rights litigation in improving access to reproductive health care and achieving reductions in maternal mortality
Source: BMC Pregnancy Childbirth. 2017 Nov 8;17(Suppl 2):367. doi: 10.1186/s12884-017-1496-0 (PMC5688491; doi:10.1186/s12884-017-1496-0)
Supplement: Additional file 1: — Open peer review. (PDF 165 kb) [file 12884_2017_1496_MOESM1_ESM.pdf]

Reviewer reports

Title: The role of human rights litigation in improving access to reproductive health care and achieving reductions in maternal mortality

Reviewer 1: Lara Stemple

- Major Compulsory Revisions

1. This paper contains an interesting argument to be made concerning the benefits of litigating maternal mortality-related claims in international fora. However, there are some organizational limitations that prevent the paper from reaching its full potential. From the outset, it is not entire clear whether the paper is normative or positive. It seems at first to be a positive (descriptive) piece providing background and the history of such litigation, or if not entirely positive, it's at least giving an overview that generally lauds these developments while providing a summary of relevant cases litigated to date. But in section IV. there is a surprising shift to a deep-dive normative debate, which then seems to make an argument along the following lines, although this argument isn't explicitly articulated in the paper (so I may well be mistaken):

   Unlike other international litigation around the right to health, which is important but has significant complicating drawbacks concerning resource constraints and vertical approaches to healthcare, maternal mortality-related claims lack these drawbacks and should therefore be pursued by advocates for gender equality, maternal healthcare, and the improvement of health outcomes generally.

   I find this to be a really interesting argument, worthy of its own paper. If this is meant to be a significant thrust of the paper, I suggest that it be clearly articulated in the beginning of the paper, together with a "roadmap" that helps explain and prepare the reader for the other portions of the paper.

   In any event, the paper will be strengthened if it clearly focuses on what the real work of the paper is – reviewing what's been done and why it's been important? Or providing a perspective on which health topics should be litigated moving forward? In particular, the following paragraph raised many big and important debates as late as page 22. Such significant questions might be more provocative (or might help with framing) if presented at the beginning and then subsequently addressed earlier on. (I don't believe the last question was addressed in the paper.)

   "Is human rights litigation an appropriate mechanism for ensuring the right to health and addressing preventable maternal mortality and morbidity, and how should scarce resources be allocated in a resource poor country? Should courts have a role in resource

allocation? Does litigation favor "those who shout the loudest," i.e., the wealthy who have money and therefore access to lawyers and the courts?"

- Minor Essential Revisions

2. "Should the court still have ordered the government to provide access and subsidize HIV/AIDS treatment if the drug companies had not provided offered free medication?" This sentence has an extra word: provided/offered.

- Discretionary Revisions

3. "Over the last decade, human rights advocates have had increasing success in securing gender-appropriate health services, including access to abortion, prenatal care, attendance during childbirth, and care during the post-partum period through human rights litigation." The term "gender-appropriate health services" sounds off here and in other places in the paper. Consider instead: women's health services or gender-specific services. The word "appropriate" connotes a degree of judgment that is unduly complicating and potentially problematic: e.g., are providers deciding what is gender "appropriate" – when women (and men) should instead decide for themselves?

4. "Similarly, local advocates, often with the support of NGOs, have sought redress from state governments for violations of the right to health in domestic constitutions." This implies a distinction between advocates and NGOs, when many NGOs are comprised of local advocates. Perhaps the authors mean international NGOs?

5. "The Declaration [UDHR], however, did not include any mechanisms for monitoring or enforcement of the enumerated rights and was not binding on the signatory nations." The UDHR is UN declaration and as such does not have signatories per se. Instead it was ratified by the UN General Assembly.

6. "A majority of nations were ready to commit to the more familiar, and less controversial, civil and political rights." Consider whether this sentence elides the significant Cold War split among counties. For Soviet sphere-of-influence countries, the ICCPR was actually more controversial than the ICESCR, which was more familiar to those governments.

7. "However, although the CESCR contains the most forceful promise of the right to health, including specific references to the right to family planning and perinatal care, the CESCR only binds nations that have ratified the treaty. Thus, advocates have looked to

other treaties…" Consider whether it's worth noting that in fact most UN member states have ratified this treaty. Sentences may imply otherwise.

8. "The case of *Alyne v. Brazil* has been the subject of substantial discussion and analysis among scholars and international bodies." Consider elaborating slightly – why so substantial? Is it controversial? Groundbreaking? Subject to interpretation?

9. "….and will influence the internal review of national policies governing maternal health, access to essential health services, and access to safe, legal abortion." Consider whether this overstates the influence this case will have/has had on access to legal abortion.

10. "…due to her under existing government programs." Unclear what this is referring to.

11. Consider whether 3 pages on Ugandan decision is disproportionate to other coverage of cases in the paper, particularly concerning constitutional debates and judicial reasoning. On the other hand, perhaps this sets up the remainder of the paper's substance.

12. "Five years later, in *Minister of Health v Treatment Action Campaign (TAC)*, the Constitutional Court intervened, reaching the opposite conclusion." I'm not sure that "opposite" is the correct word in this sentence, given that it, too, was contingent on "available resources." Consider changing to "a different conclusion."

13. "In *TAC*, it is also significant that the case against the government was brought by the Treatment Action Campaign, a local advocacy organization." It's not made clear why this is significant. Would the holding have been different if it had been brought by patients, doctors, or others?

Level of interest - An article whose findings are important to those with closely related research interests

Quality of written English - Acceptable

I declare that I have no competing interests.

Reviewer 2: Onyema Afulukwe

- Minor Essential Revisions

1. In the section titled "Using Human Rights Litigation to Promote Maternal Health," penultimate and last paragraphs: consider beginning with your thesis statement/what your key arguments or claims are and then use the other statements in those paragraphs to outline how you intend to prove your thesis. As it currently stands, what makes your article unique from other articles on this same topic isn't easily identifiable.

2. In the section titled 'Formation of the United Nations and the "International Bill of Rights,"' the last paragraph: analysis of the main difference between the ICCPR and the ICESCR still needs further revision. While the ICESCR does provide for progressive realization, the Economic, Social and Cultural Rights Committee, which is the body experts charged with its interpretation, have explained that there are minimum core obligations and obligations of equal stature with respect to the right to health which are not subject to progressive realization. This means states have an immediate duty to fulfil those obligations. One example is that of non-discrimination i.e states have an immediate obligation under the right to health to ensure health care provision is non-discriminatory. Please refer to the Committee's general comments for further information and reflect this nuanced understanding of progressive realization of ICESCR/right to health in this paragraph.

3. In the section titled, "International Covenant on Civil and Political Rights: Using Civil and Political Rights to Address the Right to Maternal Health," second paragraph: please correct typo which refers to the Human Rights Committee as the Committee on Human Rights.

4. In the section titled "Alyne v. Brazil: A Finding of Intersectional Discrimination" the last paragraph acknowledges previous analysis and studies of the case but does not cite any of those sources or explain how this particular article builds on the previous studies. It would be helpful to do so.

5. In the section titled "LC v. Peru: Litigating the Right to Abortion and the Maternal Health Exception in Peru," penultimate paragraph: statement that LC v Peru decision is binding only on Peru and persuasive in other states may not be helpful. The distinction made isn't entirely accurate—CEDAW Committee is a quasi-judicial body so its decisions are not strictly binding or enforceable on states, even Peru. Yet, when a state ratifies the CEDAW Convention, the understanding is that the state has an obligation to comply with any decisions from the Committee tasked with its interpretation. As such, the LC v Peru decision imposes obligations on all CEDAW member states—both Peru and others—and compliance is expected.

6. In the section titled "The Decision by the Delhi High Court," second paragraph, the article states that the decision being discussed has been severally written about/analyzed by scholars but doesn't provide any citations or mention how this article builds on previous work. It would be useful to do so.

7. In the section titled "The High Court of Kenya at Nairobi: Petition No. 562 of 2012," last paragraph: the statement that "the Kenyan Court became the first court from an African country to hold the government accountable for preventable maternal death" needs to be further clarified since this case was not directly a maternal death case. A more nuanced conclusion would help.

Further, the statement that the court "ordered the government to provide free, accessible and non-discriminatory public health care to (pregnant) women, irrespective of the country's scarce resources or limited budget" needs to be revisited. The court did not issue an order on this. Consider re-reading the case and reflecting what the court determined regarding free maternal health care services.

Lastly, the article discusses this case from the specific lens of access to free maternal health care services. However, this case was a hugely groundbreaking decision for other reasons which should be reflected/mentioned.

8. In the section titled "The Health System in Uganda," the "Abuja Declaration" is mentioned without first citing its full name or providing background information on what it is/means.

- Discretionary Revisions

1. In the section titled 'Formation of the United Nations and the "International Bill of Rights,"' first and second paragraphs: these two paragraphs contain a lot of descriptive and background information on the UN and the Universal Declaration of Human Rights which aren't crucial to include. Consider using citations to refer to materials for further reading on these and use the space to discuss the key international instruments you will rely on to prove your arguments.

2. In the section titled "Covenant for Economic Social and Cultural Rights (CESCR)," third paragraph: since there's a statement that advocates need to rely on other treaties beyond the ICESCR because it is only binding on states that have ratified it, this suggests that not enough have but no proof is provided. Consider including more information on the status of ratifications to confirm this assumption. This is especially necessary since the next paragraph after the statement expressly notes the number/high level of state ratification of CEDAW and indicates that advocates are relying on CEDAW as well.

3. In section titled "CRITICAL REFLECTION: Is Human Rights Litigation the Appropriate Mechanism for Ensuring the Right To Health and Addressing Preventable Maternal Mortality?" second paragraph: the article raises an important question/argument about what role resource considerations should play in litigating or deciding right to health/maternal health violations going forward, which really is its thesis and should be prioritized from the start. This is what distinguishes/could distinguish this article from previous work and could add a body of knowledge that is not yet adequately addressed. My earlier comment about balancing the article's statements about the progressive realization of the right to health with the existence

of minimum core obligations which impose immediate obligations would be relevant to this question as well.

Quality of written English - Acceptable

I declare that I have no competing interests.

Response to reviews

Comments from Supplement Managing Editor Dr. Paula Tavrow:

Dear Dr. Dunn,

This paper is potentially very valuable. In addition to the comments of the two reviewers, I would like to offer the following feedback:

1. **I think this paper would be strengthened if it kept very close to the title—in other words, if it focused on discussing the strengths and limitations of using human rights litigation to improve access and decrease maternal mortality. The audience for the paper will be mostly in the public health field, so it would be valuable for them to appreciate the role of litigation and advocacy in achieving reductions in MMR. In other words, to what extent can lawyers be partners in the effort…and how far they can go.**

   Thank you for this feedback. We have made this the focus of the paper. For each of the cases presented, we discuss changes that have been made to improve maternal health in each country as a result of the cases. In the concluding thought section we provide specific strengths and limitations, in addition to the analysis that is integrated into each case study. We also integrate the value of collaborations in each case study and end the paper with ways in which the public health and legal communities can collaborate in a way that is interdisciplinary and complements the strengths of each field.

2. **To this end, I would remove the long lead-in to the paper discussing the problem of MMR and jump right into the main subject of the paper, which is whether litigation can make a difference. I believe it would be most valuable to know just how far litigation has been able to push the issue, what restricts its impact, and potentially promising new avenues for future litigators.**

   We agree and have restructured the introduction to the paper. The introduction discussed the use of human rights litigation to improve maternal health generally and then provides a roadmap for our paper.

   With regards to the background on global maternal mortality, we felt that it was valuable to keep some key facts regarding the scale of the issue. We have included this in our global priorities section. We agree that the global maternal mortality section was too long and have kept this section shorter, focusing on the global goals to reduce maternal mortality and improve access to reproductive health services. In addition, we have integrated reproductive health services into the health background as some of our cases are not maternal deaths but address other reproductive health issues, such as access to abortion services.

We have addressed whether litigation can push the issues and its limitations in each of the case studies and the concluding thoughts.

3. **I asked one of our reviewers, Lara Stemple, to further expound on her comments (in other words, what direction she would prefer the paper followed) and she elaborated as follows:**
   "I think a normative piece that focuses on advocacy strategy would be a much more significant contribution. One often hears simple arguments along the lines of "cases concerning [insert pet cause] are important to bring." It's much more novel to hear an argument that cases concerning maternal healthcare are arguably more strategic than other health cases for the reasons they give in the paper. This analysis could be built up a little more and the background could be trimmed (though some is helpful because the journal will have mostly non-lawyer readers) so as to leave only that which is necessary to examine the particular moment we're now at in terms of litigating health claims at the international level. (Answer, as I gather from the paper: important groundwork has been laid, some health cases raise difficult questions with no clear answers vis a vis the role of courts to dictate spending, the problems with siloed approaches, but one particular kind of case avoids some of these pitfalls and should be pursued with great vigor: maternal health related claims). In short, the paper just needs more traction that takes it directly from the powerful facts at the beginning about the scope of the problem to the main thrust of the paper. As it is, it meanders a bit too much before getting to the punch of the proposed solution. (Unless I misunderstand their intention, which may be to write a descriptive paper about the history of this litigation that just happens to have some commentary as well.) I'd be happy to have a call with Jennifer or others if you think it'd be helpful! I really believe in the project and think it could be a real contribution."

   While we found the normative paper about vertical programming and the importance of maternal health litigation specifically important and interesting as well, we decided that, for the purposes of this particular paper, we would focus more on the impact of the cases. We have added an impact section to each of the cases presented and a thorough analysis of the value of litigation. We believe that we have a more refined argument now. In addition, we have cut down a substantial amount of introductory information to make the paper less descriptive.

4. **I did not really understand how litigation strengthens women's "voice and agency." If the authors want to make this case, they need to define their terms and be specific on how exactly this is occurring.**

   We have deleted this argument as we agree that it was not backed up with evidence, examples, or defining terms. We have added analysis in our concluding thoughts on how successful decisions proclaim that women's lives matter.

5. **Overall, I think that this paper represents a good first draft that now needs some careful tightening and revision. In the end, the conclusions would be best if they summarized the main points about progress made using litigation and what litigators could do next (and/or how the public health community could make better use of legal strategies to achieve better access to care).**

   Thank you. As discussed above, we made substantial changes to the paper. We have discussed the impact/progress made using litigation in each case example and summarized the impact in our concluding thoughts. In addition, we have also integrated the role of collaboration of the public health and legal communities and how litigation can be leveraged in each of the case studies and our concluding thoughts.

Reviewer 1: Lara Stemple

- Major Compulsory Revisions

1. **This paper contains an interesting argument to be made concerning the benefits of litigating maternal mortality-related claims in international fora. However, there are some organizational limitations that prevent the paper from reaching its full potential. From the outset, it is not entire clear whether the paper is normative or positive. It seems at first to be a positive (descriptive) piece providing background and the history of such litigation, or if not entirely positive, it's at least giving an overview that generally lauds these developments while providing a summary of relevant cases litigated to date. But in section IV. there is a surprising shift to a deep-dive normative debate, which then seems to make an argument along the following lines, although this argument isn't explicitly articulated in the paper (so I may well be mistaken):**

   Unlike other international litigation around the right to health, which is important but has significant complicating drawbacks concerning resource constraints and vertical approaches to healthcare, maternal mortality-related claims lack these drawbacks and should therefore be pursued by advocates for gender equality, maternal healthcare, and the improvement of health outcomes generally.

   I find this to be a really interesting argument, worthy of its own paper. If this is meant to be a significant thrust of the paper, I suggest that it be clearly articulated in the beginning of the paper, together with a "roadmap" that helps explain and prepare the reader for the other portions of the paper.

In any event, the paper will be strengthened if it clearly focuses on what the real work of the paper is – reviewing what's been done and why it's been important? Or providing a perspective on which health topics should be litigated moving forward? In particular, the following paragraph raised many big and important debates as late as page 22. Such significant questions might be more provocative (or might help with framing) if presented at the beginning and then subsequently addressed earlier on. (I don't believe the last question was addressed in the paper.)

"Is human rights litigation an appropriate mechanism for ensuring the right to health and addressing preventable maternal mortality and morbidity, and how should scarce resources be allocated in a resource poor country? Should courts have a role in resource allocation? Does litigation favor "those who shout the loudest," i.e., the wealthy who have money and therefore access to lawyers and the courts?"

We completely agree and greatly appreciate your comments. We have changed the structure of our paper and decided to split the arguments up into multiple different papers. For the purpose of this particular paper, we have removed the vertical programming/resource allocation argument and focused on the impact of the cases and how the public health community can leverage litigation. In addition, we discussed the strengths and limitations of the paper. Thank you for your comments as they helped us focus in on specific arguments rather than trying to fit everything in one paper.

We have also added a roadmap to our paper which we hope is helpful to the readers in understanding the direction of our paper. Thanks for this suggestion.

- Minor Essential Revisions

2. **"Should the court still have ordered the government to provide access and subsidize HIV/AIDS treatment if the drug companies had not provided offered free medication?" This sentence has an extra word: provided/offered.**

   Thank for noticing this. We have removed that sentence from our paper as it is part of the vertical programming argument.

- Discretionary Revisions

3. **"Over the last decade, human rights advocates have had increasing success in securing gender-appropriate health services, including access to abortion, prenatal care, attendance during childbirth, and care during the post-partum period through human rights litigation." The term "gender-appropriate health services" sounds off here and in other places in the paper. Consider instead: women's health services or gender-specific services. The word "appropriate" connotes a degree of judgment that is unduly complicating and potentially problematic: e.g., are providers deciding**

what is gender "appropriate" – when women (and men) should instead decide for themselves?

Thank for this comment and we agree. We have removed the term "gender appropriate health services" from our paper and have used "increasing access to reproductive health services and improving maternal health" throughout most of the paper.

4. **"Similarly, local advocates, often with the support of NGOs, have sought redress from state governments for violations of the right to health in domestic constitutions." This implies a distinction between advocates and NGOs, when many NGOs are comprised of local advocates. Perhaps the authors mean international NGOs?**

   The sentence has been removed from our final paper. However, you have noticed a key point that we also noticed about our inconsistent use of "advocates" and incorrectly distinguishing them from the public health community. To remedy this, we have made sure to be very clear in each circumstance to use civil society organizations, advocates, public health community, lawyers, or another appropriate term to ensure that it is clear who we are referring to.

5. **"The Declaration [UDHR], however, did not include any mechanisms for monitoring or enforcement of the enumerated rights and was not binding on the signatory nations." The UDHR is UN declaration and as such does not have signatories per se. Instead it was ratified by the UN General Assembly.**

   In order to cut down our introduction, we have removed this information, but thank you for noticing this.

6. **"A majority of nations were ready to commit to the more familiar, and less controversial, civil and political rights." Consider whether this sentence elides the significant Cold War split among counties. For Soviet sphere-of-influence countries, the ICCPR was actually more controversial than the ICESCR, which was more familiar to those governments.**

   In order to cut down our introduction on human rights treaties, we have removed this information. We have removed the section on ICESCR and cut down the ICCPR section.

7. **"However, although the CESCR contains the most forceful promise of the right to health, including specific references to the right to family planning and perinatal care, the CESCR only binds nations that have ratified the treaty. Thus, advocates have looked to other treaties…" Consider whether it's worth noting that in fact most UN member states have ratified this treaty. Sentences may imply otherwise.**

   We have deleted the entire section on CESCR as it was not relevant to the case studies presented.

8. **“The case of *Alyne v. Brazil* has been the subject of substantial discussion and analysis among scholars and international bodies.” Consider elaborating slightly – why so substantial? Is it controversial? Groundbreaking? Subject to interpretation?**

   On page 9, we have elaborated on this and added citations. The sentence now reads: “It has also been the subject of substantial discussion and analysis among scholars [30,35–37] as a groundbreaking model for holding governments accountable for gender-based discrimination in health care and preventable maternal mortality under international law.”

9. **“….and will influence the internal review of national policies governing maternal health, access to essential health services, and access to safe, legal abortion.” Consider whether this overstates the influence this case will have/has had on access to legal abortion.**

   We have deleted this particular sentence. We now discuss the impact of the cases in more depth, including how Peru changed their abortion guidelines in 2014.

10. **“…due to her under existing government programs.” Unclear what this is referring to.**

    Thank for noticing this. The sentence now reads “Fatima made several visits to government health facilities and shelters and was denied medical care and other assistance guaranteed under existing government programs” on page 16.

11. **Consider whether 3 pages on Ugandan decision is disproportionate to other coverage of cases in the paper, particularly concerning constitutional debates and judicial reasoning. On the other hand, perhaps this sets up the remainder of the paper’s substance.**

    We agree. It did set up our vertical programming argument well but it was much too long. Since we have cut the vertical programming section for the purposes of this paper, the Uganda section has been refined and modified to fit the structure of the rest of the cases. While it is still 3.5 pages, it provides a different purpose and more analysis has been provided. We feel that there is an even distribution of information and analysis for each case now.

12. **“Five years later, in *Minister of Health v Treatment Action Campaign (TAC)*, the Constitutional Court intervened, reaching the opposite conclusion.” I’m not sure that “opposite” is the correct word in this sentence, given that it, too, was contingent on “available resources.” Consider changing to “a different conclusion.”**

    We have also deleted this section as a result of changing the structure and refining our arguments, but thank you for this suggestion.

13. **“In *TAC*, it is also significant that the case against the government was brought by the Treatment Action Campaign, a local advocacy organization.” It’s not made**

clear why this is significant. Would the holding have been different if it had been brought by patients, doctors, or others?

We have also deleted this section.

Reviewer 2: Onyema Afulukwe

- Minor Essential Revisions

1. In the section titled "Using Human Rights Litigation to Promote Maternal Health," penultimate and last paragraphs: consider beginning with your thesis statement/what your key arguments or claims are and then use the other statements in those paragraphs to outline how you intend to prove your thesis. As it currently stands, what makes your article unique from other articles on this same topic isn't easily identifiable.

The first paragraph of this section has been edited and moved to the introduction of our paper. The rest of the section has been removed from the paper. However, we agree that we need to make our arguments more clear from the beginning and, therefore, provided a roadmap for our paper in the introduction.

2. In the section titled 'Formation of the United Nations and the "International Bill of Rights,"' the last paragraph: analysis of the main difference between the ICCPR and the ICESCR still needs further revision. While the ICESCR does provide for progressive realization, the Economic, Social and Cultural Rights Committee, which is the body experts charged with its interpretation, have explained that there are minimum core obligations and obligations of equal stature with respect to the right to health which are not subject to progressive realization. This means states have an immediate duty to fulfil those obligations. One example is that of non-discrimination i.e states have an immediate obligation under the right to health to ensure health care provision is non-discriminatory. Please refer to the Committee's general comments for further information and reflect this nuanced understanding of progressive realization of ICESCR/right to health in this paragraph.

Due to the revisions we have made to the paper, this section has been deleted. In addition, we have deleted the ICESCR section as it is not cited by our presented case studies. Thank you for pointing out this important revision.

3. In the section titled, "International Covenant on Civil and Political Rights: Using Civil and Political Rights to Address the Right to Maternal Health," second paragraph: please correct typo which refers to the Human Rights Committee as the Committee on Human Rights.

We have made this change and ensured that it is accurate throughout the paper.

4. In the section titled "Alyne v. Brazil: A Finding of Intersectional Discrimination" the last paragraph acknowledges previous analysis and studies of the case but does not cite any of those sources or explain how this particular article builds on the previous studies. It would be helpful to do so.

On page 9, we have elaborated on this and added citations. The sentence now reads: "It has also been the subject of substantial discussion and analysis among scholars [30,35–37] as a groundbreaking model for holding governments accountable for gender-based discrimination in health care and preventable maternal mortality under international law."

5. In the section titled "LC v. Peru: Litigating the Right to Abortion and the Maternal Health Exception in Peru," penultimate paragraph: statement that LC v Peru decision is binding only on Peru and persuasive in other states may not be helpful. The distinction made isn't entirely accurate—CEDAW Committee is a quasi-judicial body so its decisions are not strictly binding or enforceable on states, even Peru. Yet, when a state ratifies the CEDAW Convention, the understanding is that the state has an obligation to comply with any decisions from the Committee tasked with its interpretation. As such, the LC v Peru decision imposes obligations on all CEDAW member states—both Peru and others—and compliance is expected.

We have removed this information as we didn't feel it was necessary to include in our refined paper. However, we appreciate clarifying this distinction.

6. In the section titled "The Decision by the Delhi High Court," second paragraph, the article states that the decision being discussed has been severally written about/analyzed by scholars but doesn't provide any citations or mention how this article builds on previous work. It would be useful to do so.

On page 16, we have added citations and the sentence now reads: "The *Laxmi Mandal* decision has been the subject of substantial review by legal scholars [54,56] and has influenced subsequent decisions in India and domestic courts in other countries [11,13,14,56,57]."

7. In the section titled "The High Court of Kenya at Nairobi: Petition No. 562 of 2012," last paragraph: the statement that "the Kenyan Court became the first court from an African country to hold the government accountable for preventable maternal death" needs to be further clarified since this case was not directly a maternal death case. A more nuanced conclusion would help.

We absolutely agree. We have decided to remove this case to focus on the other cases presented. However, we have changed our paper to include access to reproductive health services and maternal death to make sure we are making the distinction that not all of the cases included maternal death (both cases in Peru and *Jaitun* case in India)

Further, the statement that the court "ordered the government to provide free, accessible and non-discriminatory public health care to (pregnant) women, irrespective of the country's scarce resources or limited budget" needs to be revisited. The court did not issue an order on this. Consider re-reading the case and reflecting what the court determined regarding free maternal health care services.

We ended up deleting the case for a variety of reasons, but we are very much interested in the case and we appreciate the expertise that you have provided.

Lastly, the article discusses this case from the specific lens of access to free maternal health care services. However, this case was a hugely groundbreaking decision for other reasons which should be reflected/mentioned.

We completely agree. However, we have deleted the case for the purposes of this paper.

8. In the section titled "The Health System in Uganda," the "Abuja Declaration" is mentioned without first citing its full name or providing background information on what it is/means.

To refine and simplify the Ugandan section, we have cut this information. However, we appreciate that you pointed this out and definitely agree that it needed more clarification.

- Discretionary Revisions

1. In the section titled 'Formation of the United Nations and the "International Bill of Rights,"' first and second paragraphs: these two paragraphs contain a lot of descriptive and background information on the UN and the Universal Declaration of Human Rights which aren't crucial to include. Consider using citations to refer to materials for further reading on these and use the space to discuss the key international instruments you will rely on to prove your arguments.

We completely agree and have deleted this section. We have focused on explaining CEDAW and ICCPR, as those are the instruments used in our cases presented.

2. In the section titled "Covenant for Economic Social and Cultural Rights (CESCR)," third paragraph: since there's a statement that advocates need to rely on other treaties beyond the ICESCR because it is only binding on states that have ratified it, this suggests that not enough have but no proof is provided. Consider including more information on the status of ratifications to confirm this assumption. This is especially necessary since the next paragraph after the statement expressly notes the number/high level of state ratification of CEDAW and indicates that advocates are relying on CEDAW as well.

We have deleted the section on CESCR as it was not used in any of the case studies we presented. We have also deleted the number of states that have ratified the treaties in order to refine and cut down on some of the information in the international law section.

3. In section titled "CRITICAL REFLECTION: Is Human Rights Litigation the Appropriate Mechanism for Ensuring the Right To Health and Addressing Preventable Maternal Mortality?" second paragraph: the article raises an important question/argument about what role resource considerations should play in litigating or deciding right to health/maternal health violations going forward, which really is its thesis and should be prioritized from the start. This is what distinguishes/could distinguish this article from previous work and could add a body of knowledge that is not yet adequately addressed. My earlier comment about balancing the article's statements about the

progressive realization of the right to health with the existence of minimum core obligations which impose immediate obligations would be relevant to this question as well.

We think this is an excellent and interesting point about the progressive realization and would like to address that in future analysis of our resource allocation argument. For the purposes of this paper, we have decided to remove the resource allocation part and formulate it into a separate paper. Based on reviewer comments, we understand that this is an important argument and we hope to write another paper that gives us the space to fully develop that argument. We felt that it was best for this paper to focus on the impact of each case presented, the strengths and limitations, and how public health and legal communities can leverage litigation. We hope you find this newly refined paper and analysis interesting as well.

Reviewer reports – 2nd round

Reviewer 2: Onyema Afulukwe

Please find the manuscript attached with my few comments. They've been added directly into the manuscript using comment boxes but am happy to provide them in a different way, if preferable.

Beyond the comments I've already made, I would just add that this new version of the manuscript is more streamlined. It's focus is clear--the pros and cons of using litigation to advance reproductive health and maternal health. This is great. I also understand that the audience will be largely non-lawyers and as such an article that provides examples of how litigation has been used to better protect women's reproductive health would be beneficial.

Otherwise, if the intended audience would have been lawyers, I would have suggested going beyond providing these instances of litigation since they are already well known to most human rights lawyers/advocates. Nonetheless, my final comment--in the comment box--still suggests mentioning some opportunities that could still be explored in the future so that the article isn't only reiterating what has been done but also suggesting what should/could be done moving forward.

Reviewer 1: Lara Stemple

I read the paper, which has changed substantially! Kudos to the authors for such a herculean effort! It's a good, case-intensive overview of litigation in various fora related to maternal mortality, with reasonably supported claims about HR litigation as a useful tool to address this problem. I view it as absolutely worthy of publication. The article will be of interest to those who'd like an overview of such cases, and it reads very clearly in that regard.

In my view, the truly novel claim in the earlier version -- that cases concerning maternal healthcare are arguably more strategic than other health cases for the reasons they had given – is no longer emphasized, which is too bad (I think this interesting claim should be its own paper!). Instead the paper is now more like many articles which make a familiar argument along the lines of "cases concerning [insert pet cause] are important to bring." These arguments are fine and even important, just not that novel. But arguably novel enough for a non-legal audience specifically interested in maternal healthcare. Plus, the authors do a good job of explaining the limits of HR litigation and the need to use it as part of a broader civil society strategy.

One small point is that, when enumerating the limits, they might also note that very, VERY few people ultimately access the international fora, so it's not like a legal aid clinic, but rather an impact strategy. This is obvious to lawyers, but I find that non-lawyers are sometimes usefully reminded that these courts serve nearly no one in terms of actual access to justice through them.

Overall, I don't think the paper requires much more work at all, for what it is aiming to do. Here are a few items I flagged for fact-checking.

"As a result of a concerted effort by these organizations to bring maternal health and mortality into the international human rights agenda, the maternal mortality ratio decreased by approximately 44 percent between 1990 and 2015, from 385 to 216 deaths per 100,000 live births [16]." This overstates the causal connection between the human rights agenda and the entire decrease in maternal mortality, which has lots of medical, social, public health, and other causal factors.

"This promise includes ensuring that women have the right to vote and hold elected office, the right to pursue an education, the right to own property, and the right to health [27]." CEDAW does include some specific health provisions, but not the "right to health" per se, which is a term of art in HR discourse.

"As the first case of an international treaty body to hold a government accountable for preventable maternal death, Alyne v. Brazil brought global attention to the issue and influenced both international [4] and domestic courts [15] charged with reviewing sex, race and socioeconomic discrimination, and related health system disparities." I wonder if these cases actually mention Alyne (I don't think so) and whether they address race at all (I don't think so, but that's probably ok because authors are not directly claiming this). Worth making sure the sources support the claim here.

I hope this is helpful. Feel free to forward any or all of this that may be of use. And if you do: congratulations to the authors for working so hard to create a clear, readable, and useful paper – well done!!! Cheers,
